# Identification of SYK inhibitor, R406 as a novel senolytic agent

**DOI:** 10.18632/aging.103135

**Published:** 2020-05-07

**Authors:** Hyun-Ji Cho, Eun Jae Yang, Joon Tae Park, Jae-Ryong Kim, Eok-Cheon Kim, Kyong-Jin Jung, Sang Chul Park, Young-Sam Lee

**Affiliations:** 1 Well Aging Research Center, DGIST, Daegu 42988, Korea; 2 Department of Medicine, Catholic University of Daegu School of Medicine, Daegu 42472, Korea; 3 Department of New Biology, DGIST, Daegu 42988, Korea; 4 Division of Life Sciences, College of Life Sciences and Bioengineering, Incheon National University, Incheon 22012, Korea; 5 Department of Biochemistry and Molecular Biology, Smart-Aging Convergence Research Center, College of Medicine, Yeungnam University, Daegu 42415, Korea; 6 Department of Molecular Medicine, Chonnam National University Medical School, Gwangju 58128, Korea; 7 The Future Life and Society Research Center, Chonnam National University, Gwangju 58128, Korea

**Keywords:** cellular senescence, senolytics, apoptosis, FAK, p38

## Abstract

The selective removal of senescent cells by senolytics is suggested as a potential approach to reverse aging and extend lifespan. Using high-throughput screening with replicative senescence of human diploid fibroblasts (HDFs), we identified a novel senolytic drug R406 that showed selective toxicity in senescent cells. Using flow cytometry and caspase expression analysis, we confirmed that R406 caused apoptotic cell death along with morphological changes in senescent cells. Interestingly, R406 altered the cell survival-related molecular processes including the inhibition of phosphorylation of the focal adhesion kinase (FAK) and p38 mitogen-activated protein kinase (MAPK) in senescent cells. This pattern was not observed in other known senolytic agent ABT263. Correspondingly, apoptotic cell death in senescent cells was induced by simultaneously blocking the FAK and p38 pathways. Taken together, we suggest that R406 acts as a senolytic drug by inducing apoptosis and reducing cell attachment capacity.

## INTRODUCTION

Aging is a progressive physiological change over time and is mainly associated with the decline of biological functions and senescence in an organism [1]. The loss of biological function in cells, the basic units of tissue and organ, can be caused by cellular stresses, including genomic instability, impaired proteostasis, and dysfunctional subcellular organelles, all of which provoke the cessation of cell division and cellular senescence [2–5]. Senescent cells have a variety of characteristics such as the irreversible arrest of the cell cycle, resistance to apoptosis, increasing activity of senescence-associated β-galactosidase (SA-β-gal), and secretion of a set of various cytokines involving chronic inflammation and structure remodeling, known as senescence-associated secretory phenotypes (SASPs) [2, 3, 6–9]. Although the transient appearance of cellular senescence plays an essential role in tissue remodeling, efficient immune clearance during embryonic development, and after an injury [10–12], a loss of proliferation capacity and SASP secretion in chronic senescent cells per se affects the repair of impaired tissues. Therefore, cellular senescence is suggested to be strongly linked with age-associated tissue damage and diseases. Recent evidence also points to close linkage between cellular senescence and tissue dysfunction. Transplanting senescent cells induced physical dysfunction in young mice [13]. In contrast, the clearance of p16^Ink4a^-positive senescent cells extends lifespan in an aging-induced transgenic mouse model, INK-ATTAC mice [14], and a mouse model of a human progeroid syndrome, Ercc1-/ mice [15].

Senotherapeutics, which are small molecules targeting cellular senescence for delaying age-related processes or diseases, have been developed mainly for two uses: one is senomorphic medicines, which suppress cellular senescence and induce reverse senescence phenotypes, and the other is senolytic drugs, which induce cytotoxicity selectively in senescent cells [16]. The combination of dasatinib and quercetin are the first senolytic agents effective in the removal of ionizing radiation-induced senescent cells *in vitro* and alleviating age-related symptoms in the progeroid Ercc1−/Δ mouse model *in vivo* [17]. Emerging evidence has demonstrated that senolytic agents alleviate various age-related conditions in mice, including age-associated vascular phenotypes [18], metabolic dysfunction [19], and osteoarthritis [20], and even affect senescence-related dysfunctions in human [21].

Major classes of senolytic drugs typically focus on inhibiting pro-survival pathways or triggering pro-apoptosis signaling in senescent cells. The combination of dasatinib and quercetin, which reduced p21, PAI-2, and BCL-xL [17], and Navitoclax (ABT263), which targets the Bcl-2 family [22], belong to this class of senolytics. In other classes, the mimicry of forkhead box protein O4 (FOXO4) peptide selectively disrupted the p53-FOXO4 interaction, which induced p53-dependent apoptosis in senescent cells [23]. Recently, a HSP90 inhibitor was identified as a novel class of senolytic drugs that downregulated the phosphorylation of PI3K/AKT, an anti-apoptotic factor [15]. Despite intensified efforts to develop drugs targeting senescent cells, however, the number of senolytic agents is still limited in comparison with the number of drugs against other age-related diseases like cancer or fibrosis. Finding a novel senotherapeutic would expand the spectrum of efficacy on various types and stages of cellular senescence.

In this study, using high-throughput screening (HTS) to measure the variation of cell proliferation and reactive oxygen species (ROS) levels, we identified a novel senolytic agent R406, also known as tamatinib. This agent was effective in the replicative senescence (RS) model of diploid human dermal fibroblasts (HDFs). R406 induced the caspase-9-mediated intrinsic apoptotic pathway, similar to other known senolytic drugs; however, R406 did not significantly change the level of Bcl-2 family in senescent cells. Alternatively, R406 inhibited the phosphorylation of focal adhesion kinase (FAK) as well as p38 mitogen-activated protein kinase (MAPK), which both regulate cell survival. Our results demonstrate that R406 is a new class of senolytics that targets multiple regulatory pathways for senescent cell survival.

## RESULTS

### R406 reduces cell viability in senescent HDFs

**Figure 1:**
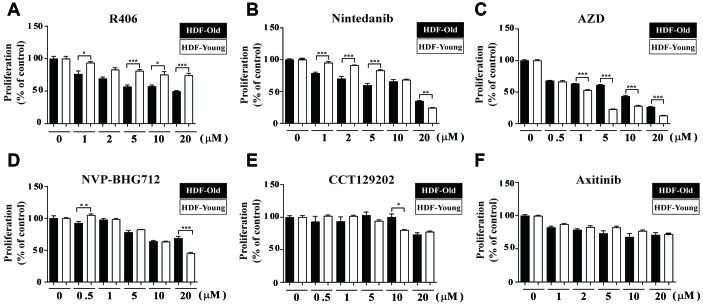
**R406 reduces viability of senescent HDFs.** Senescent HDFs (HDF-Old) and non-senescent HDFs (HDF-Young) were treated with (**A**) R406 (1, 2, 5, 10, 20 μM), (**B**) Nintedanib (1, 2, 5, 10, 20 μM), (**C**) AZD (0.5, 1, 5, 10, 20 μM), (**D**) NVP-BHG712 (0.5, 1, 5, 10, 20 μM), (**E**) CCT129202 (0.5, 1, 5, 10, 20 μM), and (**F**) Axitinib (1, 2, 5, 10, 20 μM) for one day, and then CCK-1 assays were conducted to investigate cell viability. n = 8, *p<0.05, **p<0.01, ***P<0.001.

In our previous studies, we evaluated the ability to restore senescent fibroblasts in the RS model using HTS with a library containing 355 kinase inhibitors [24, 25]. From these results, we selected candidates for their senolytic activity based on inducing cytotoxicity, increasing ROS levels, or both (Supplementary Table 1). Next, we selected out second candidate compounds by reviewing the publications on the chosen drugs initially regarding cell physiologies (e.g., senolytic effect, apoptosis, and cell death) and side effects in pre-clinical studies (e.g., high toxicity, diarrhea, fever, rash, etc.). Then, based on the CCK-1 assay, we assessed the differential cytotoxicity of the remaining candidates depending on the state of cellular senescence in HDFs (Supplementary Figure 1). Among these, R406, an FDA-approved Syk inhibitor, was found to exhibit higher cytotoxicity in senescent HDFs than in non-senescent cells over the tested range of concentration (from 1 to 20 μM; Figure 1A). Nintedanib, a tyrosine kinase inhibitor, also showed senolytic effects at lower concentrations (from 1 to 5 μM) but was toxic in higher concentration (20 μM), regardless of the senescent state (Figure 1B). Other drug candidates were not suitable as senolytic drugs due to either non-selective cytotoxicity (NVP-BHG712, an Ephrin type-B receptor 4 inhibitor; AZD, an ALK inhibitor; CCT129202, an aurora kinase inhibitor; and axitinib, a tyrosine kinase inhibitor; Figure 1C–1F); or were drugs that had no cytotoxic effect (bosutinib, an Src inhibitor; and selumetinib, a MEK inhibitor; Supplementary Figure 2A and 2B). In addition, we further confirmed R406-induced cytotoxicity by Hoechst 33342 staining, because CCK-1-based cell viability assay could reflect cell metabolic activity, which might be influenced by kinase inhibitors. Similar to the CCK-1 assay, the fluorescence levels of senescent HDFs were reduced by R406 in a dose dependent manner (Supplementary Figure 2C). Our results demonstrated that R406 has cytotoxicity selectively in senescent fibroblasts in the RS model and, subsequent experiments were performed using R406 concentration of 10 μM.

### R406 induces apoptotic cell death

**Figure 2:**
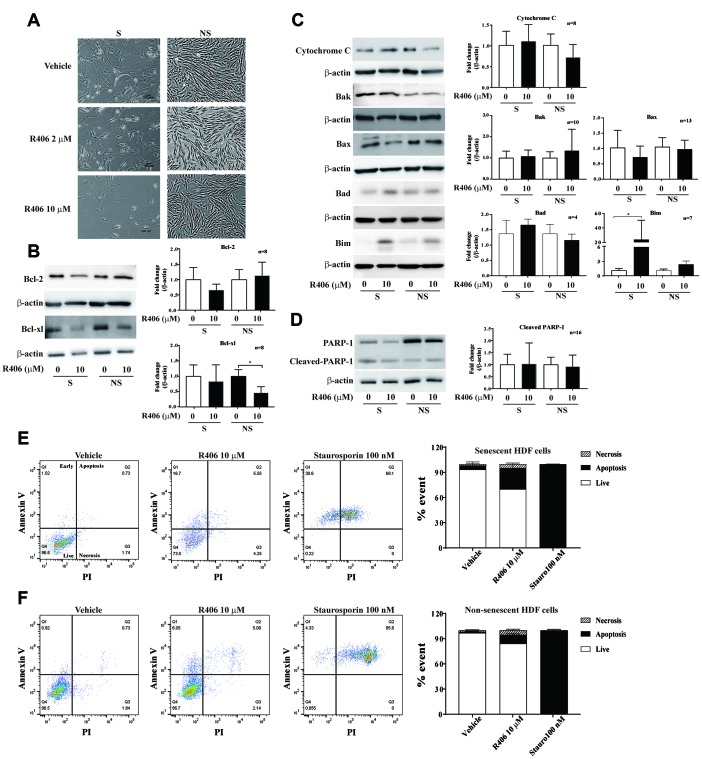
**R406 induces apoptotic cell death in senescent HDFs.** (**A**) Cell morphological change. Senescent (S) HDFs were plated in 6-well plates at a density of 6×10^4^ cell per well and non-senescent (NS) HDFs were plated in 6-well plates at a density of 8×10^4^ cell per well. And then, the HDFs were treated with DMSO or R406 (2, 10 μM) for one day. Images were randomly captured by inverted microscopy (scale bar: 100 μm). (**B**–**D**) Senescent and non-senescent HDFs were treated with DMSO or R406 (10 μM) for one day and then western blot assays were conducted to determine changing expression levels of apoptosis regulating factors. (**E**, **F**) Senescent and non-senescent HDFs were respectively treated with DMSO, R406 (10 μM), and staurosporine (100 nM) for one day and apoptosis assays with Annexin V/PI staining were conducted.

**Figure 3:**
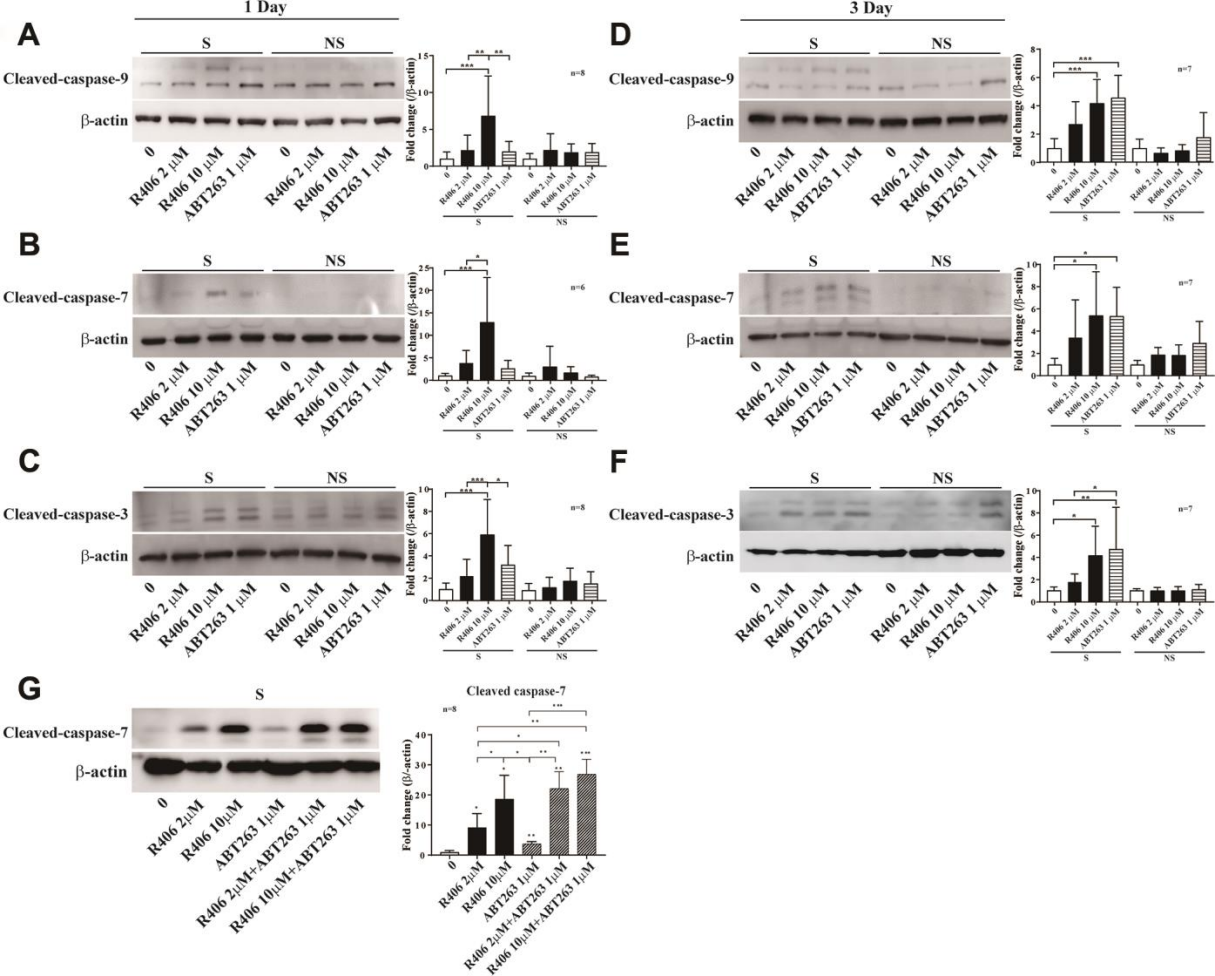
**R406 induces cleavage of caspases in senescent HDFs.** (**A**–**C**) Senescent (S) and non-senescent (NS) HDFs were treated with DMSO, R406 (2, 10 μM), and ABT263 (1 μM) for one day and then western blot assays using anti-caspase antibodies were conducted to determine senolytic effect by apoptosis. (**D**–**F**) Senescent and non-senescent HDFs were treated with DMSO, R406 (2, 10 μM), and ABT263 (1 μM) for three days and then monitored the level of cleaved-caspases by western blot analyses. (**G**) Senescent HDFs were respectively treated with single or combination of R406 and ABT263 for one day and then western blot assay with anti-caspase-7 antibody was conducted to determine apoptosis. *p<0.05, **p<0.01, ***P<0.001.

Senescent cells demonstrate elevated expression of anti-apoptotic and pro-survival factors [15, 17, 22]. Typical senolytic agents inhibit cell viability by modulating expression of these factors, particularly Bcl-2 family [26]. Therefore, we investigated whether the cytotoxic effect of R406 is caused by the induction of apoptosis. R406 induced cell shrinkage in senescent HDFs compared to non-senescent HDFs, and decreased cell number of senescent HDFs. The non-senescent HDFs showed only slightly delayed cell growth with R406 (Figure 2A). Unexpectedly, R406-dependent downregulation of Bcl-2 and Bcl-xl expression was not significant in senescent HDFs as compared to non-senescent HDFs (Figure 2B). Instead, the level of Bim as well as cleaved-caspase-9, -7, and -3 were induced by R406 in senescent HDFs, though the amount of cytochrome C, Bak, Bax, and Bad were also not significantly changed (Figures 2C and 3). The levels of cleaved-caspase-8, an indicator of extrinsic apoptotic signal induction, or cleaved-PARP-1, which is a late apoptotic marker, were not changed by R406 treatment in either senescent or non-senescent cells (Supplementary Figure 3A and Figure 2D, respectively). To confirm that R406 is associated with apoptosis in a senolytic effect, we performed an apoptosis assay by flow cytometry with annexin V and propidium iodide (PI) staining after treating either a vehicle (dimethyl sulfoxide; DMSO) or 10 μM R406 for one day in senescent and non-senescent HDFs. For the analysis, staurosporine was used as a positive control of the apoptotic effect. As a expect, approximately 23% of R406 treated senescent cells were annexin V-positive compared with only 11% in the R406-treated non-senescent cells. (Figure 2E and 2F). These data indicate that R406 induces apoptotic cell death on senescent cells in the RS model.

Previous studies demonstrated that ABT263 selectively induced apoptotic cell death in ionizing radiation-induced senescent HUVECs, IMR-90 cells, and preadipocytes at the sub-micromolar concentration [22]. Moreover, ABT263 rejuvenated aged hematopoietic stem cells in mice [27]. Thus, we investigated whether R406 can induce an apoptotic effect similar to ABT263. For this purpose, we tested the concentration range at which ABT263 demonstrated a senolytic effect in our system. Contrary to our expectation, a one day exposure of ABT263 (at 0.1~10 μM) was not found to be effective in cytotoxicity in either senescent or non-senescent HDFs (Supplementary Figure 4A–4C) compared with the R406 senolytic effect induced over the same time (Figure 1). Morphological changes and the expression of cleaved-caspase-9, -3 and -7 were induced by ABT263 but only after a three-day exposure at a much higher concentration (more than 1 μM) than conventionally used (Supplementary Figure 4D–4F). Thus, subsequent experiments were performed using 1 μM of ABT263.

In our results, R406 exhibited a senolytic effect one day post-treatment (Figures 1 and 2), whereas most of reported senolytic drugs needed to be treated for two days or more on senescent cells to show effectiveness [17, 22, 28]. Thus, we examined the senolytic effects of R406 in comparison to ABT263 over an appropriate treatment time in the RS model of HDFs. For this purpose, we treated R406 (2 or 10 μM) and ABT263 (1 μM) for one or three days on HDFs. Interestingly, R406 induced greater expression of cleaved-caspase-9, -7, and -3 after one day of treatment than ABT263 did in senescent cells (Figure 3A–3C). The expression of cleaved-caspase-9 and -7 was at similar levels between R406 and ABT263 treatment after three days (Figure 3D–3F). In addition, morphological changes to cell shrinkage appeared from 3 h in R406-treated senescent cells, whereas ABT263 did not induce significant change as viewed with time-lapse cell imaging (Supplementary Video 1). The results indicate that R406 triggers a senolytic effect much faster than ABT263 or other senolytic drugs that have been studied [17, 22, 26, 28].

As in the example of the combination of dasatinib and quercetin in which senolytic effect is potentiated [17], we additionally tested the additive senolytic effect of R406 with ABT263. Interestingly, the expression of cleaved-caspase-7 was more induced by co-treating with R406 (2 μM or 10 μM) and ABT263 (1 μM) than by individual treatment of R406 or ABT263 (Figure 3G), which indicates that R406 and ABT263 might have different modes in cell death activation and R406 with combination of other senolytic drugs could potentiate senescent cell death selectively.

We next assessed the senolytic effect of R406 in stress-induced premature senescence (SIPS) model as shown that in the RS. Cellular senescence was induced by either etoposide (inducing DNA damage) or H_2_O_2_ (increasing ROS level), which was confirmed by the upregulation of cell cycle regulators p16 and p21 (Supplementary Figure 3B). R406 inhibited the cell viability of etoposide or H_2_O_2_-treated HDFs in dose-dependent manner (Supplementary Figure 3C). Moreover, R406 induced the expression of cleaved-caspase-9 and-7 in the stress-induced HDFs one day post drug treatment (Supplementary Figure 3D, 3E). The results indicate that R406 exhibits senolytic effect in various modalities of cellular senescence.

### R406-induced ROS generation is associated with senescent cell death

**Figure 4:**
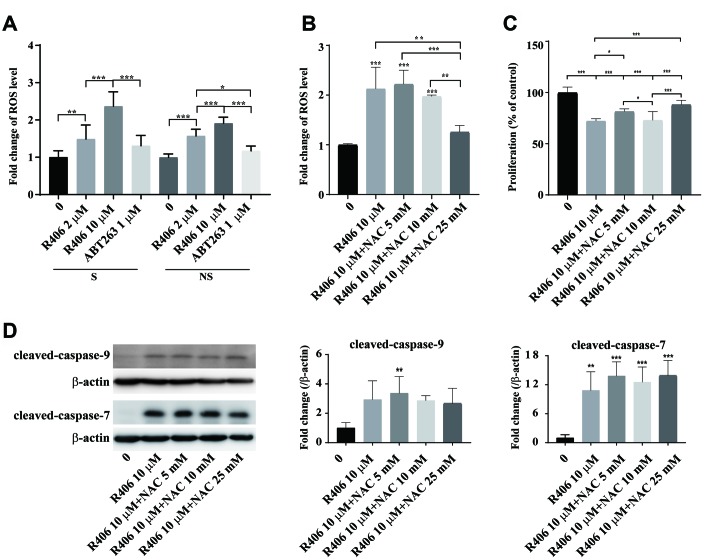
**R406 induces ROS generation.** (**A**) Senescent (S) and non-senescent (NS) HDFs were treated with DMSO or R406 (10 μM) or ABT263 (1 μM) for one day and then ROS levels were detected with DHR123 staining with normalization by DNA contents. (**B**) Senescent and non-senescent HDFs were treated with R406 in the presence or absence of NAC for one day and then ROS levels were measured after DHR123 staining by flow cytometry. (**C**) Senescent HDFs were co-treated with R406 and NAC for one day and then CCK-1 assay were conducted to investigate cell viability. n = 8 (**D**) Senescent HDFs were co-treated with R406 and NAC for one day and then western blot assays using antibodies against caspase-9, and -7 were conducted. *p<0.05, **p<0.01, ***P<0.001

Next, we examined how R406 triggers apoptosis in senescent cells. Recent studies show that R406 induces ROS-dependent apoptotic effects via Syk-dependent and independent mechanisms in glioma stem cells [29]. For that reason, we tested whether R406 induces apoptosis via ROS generation in senescent HDFs. R406 induced ROS levels in a dose-dependent manner (Figure 4A). Notably, at 10 μM of R406, higher ROS levels were observed in senescent cells than in non-senescent cells (Figure 4A). However, R406 suppressed phosphorylation of Syk regardless of cell’s senescence state (Supplementary Figure 5). In order to check the relationship between R406-mediated ROS generation and apoptosis of senescent cells, we tested the R406-induced apoptotic effect in the presence of an antioxidant, N-acetyl-L-cysteine (NAC). We confirmed that 25 mM of NAC reduced about 70% of R406-mediated ROS generation (Figure 4B). Interestingly, NAC reduced R406-induced cytotoxicity in senescent HDFs in a statistically significant manner (Figure 4C). However, the level of cleaved-caspase-9 and -7 was not changed in R406 treated senescent HDFs even in the presence of NAC (Figure 4D). These results indicate that R406-dependent ROS generation would be a factor in senescent cell death, even though it might not involve apoptosis in senescent cells.

### R406 induces a senolytic effect via dual inhibition of FAK and p38 MAPK

**Figure 5:**
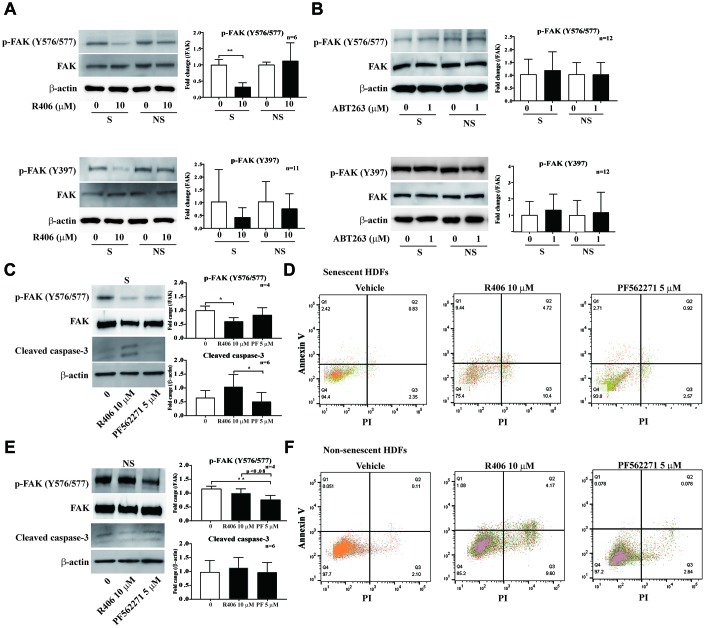
**R406 inhibits phosphorylation of FAK in senescent HDFs more than non-senescent cells.** (**A**, **B**) Senescent (S) and non-senescent (NS) HDFs were treated with R406 (10 μM, **A**), or ABT263 (1 μM, **B**) for 1 h and then western blot assays with anti-p-FAK and FAK antibodies. (**C**, **D**) Senescent HDFs were respectively treated with DMSO, R406 (10 μM), and PF562271 (5 μM) for one day and then western blot assay (anti-p-FAK and caspase-3, **C**) and apoptosis assay with Annexin V/PI staining (**D**) were conducted to determine senolytic effect. (**E**, **F**) Western blot (**E**) and apoptosis assay (**F**) with non-senescent HDFs as described in **C** and **D**. *p<0.05, **p<0.01, ***P<0.001.

Cell survival is regulated by various signal pathways that are controlled by protein kinases [30–35]. Previous studies showed that FAK, a key molecule for cell adhesion, is critical for cell survival; blocking the FAK pathway increases the loss of substrate adhesion and induction of apoptosis of anchorage-dependent cells [36, 37]. Notably, our observation regarding live cell imaging led us to hypothesize that R406 induces morphological change in senescent cells via decreased cell adhesion. To validate this hypothesis, we checked the phosphorylation of FAK after short-term exposure to senolytic drugs. R406 altered the phosphorylation of FAK levels only in senescent HDFs (Figure 5A), whereas ABT263 did not change the phosphorylation of FAK. (Figure 5B). We then examined whether R406 affects the apoptotic effect on senescent cells via FAK signaling. Senescent and non-senescent HDFs were treated with a DMSO or a FAK inhibitor, PF562771, for one day and the expression of cleaved-caspase-3 and staining of annexin V/PI measured. Contrary to our expectations, the FAK inhibitor did not induce either the cleaved-caspase-3 expression or an apoptotic effect in HDFs (Figure 5C–5F). These results imply that R406-mediated FAK dephosphorylation would be one of the critical factors for inducing senescent cell death; however, FAK inactivation would not be a sole causality for the apoptosis of senescent cells.

**Figure 6:**
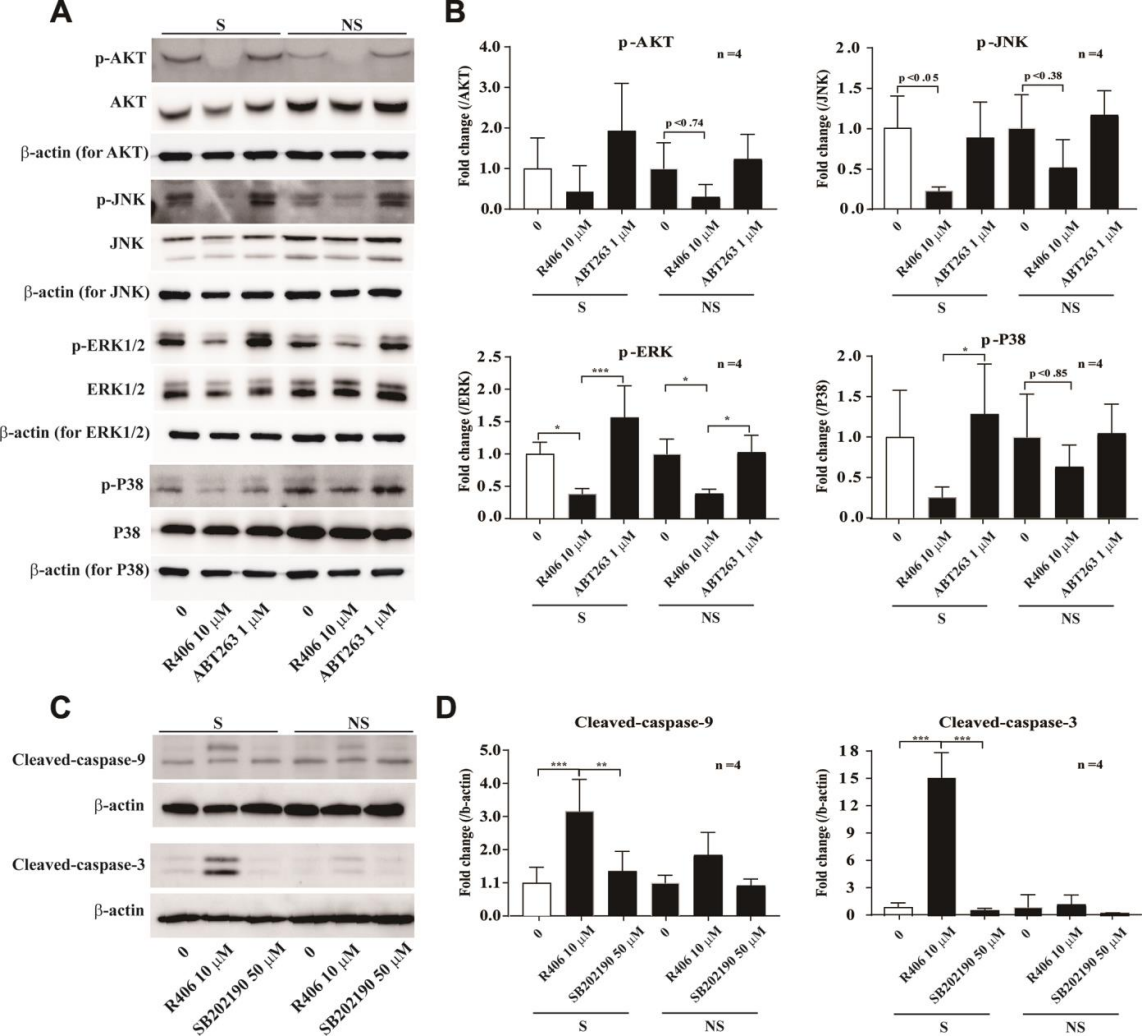
**R406 inhibits phosphorylation of p38 MAPK in senescent HDFs more than non-senescent HDFs.** (**A**, **B**) Senescent (S) and non-senescent (NS) HDFs were treated with DMSO, R406 (10 μM), and ABT263 (1 μM) for one day and western blot assays using anti-MAPKs antibodies were conducted. (**C**, **D**) Senescent HDFs were respectively treated with DMSO, R406 (10 μM), and SB202190 (p38 inhibitor, 50 μM) for one day. Then, western blot assays were conducted to determine triggering apoptosis by caspase cleavage. *p<0.05, **p<0.01, ***P<0.001.

Mitogen-activated protein kinases (ERK, JNK, and p38) and AKT mediate cell survival and death signals, which are stimulated by a diverse range of cytokine, growth factor, and chemical agents in mammalian cells [30–35]. Thus, we studied whether the senolytic effect of R406 is related to the MAPKs and AKT signaling pathways. The results showed that phosphorylation of AKT, JNK, and ERK was inhibited by R406, regardless of cellular senescence. Interestingly, p38 phosphorylation was more inhibited by R406 in senescent cells than non-senescent cells (Figure 6A, 6B). However, a p38 inhibitor, SB202190, did not stimulate cleaved-caspase-9 and -3 (Figure 6C, 6D). These results also indicate that the R406-mediated p38 dephosphorylation might be associated with senescent cell death, but p38 inactivation would not be the only reason for apoptosis of senescent cells.

**Figure 7:**
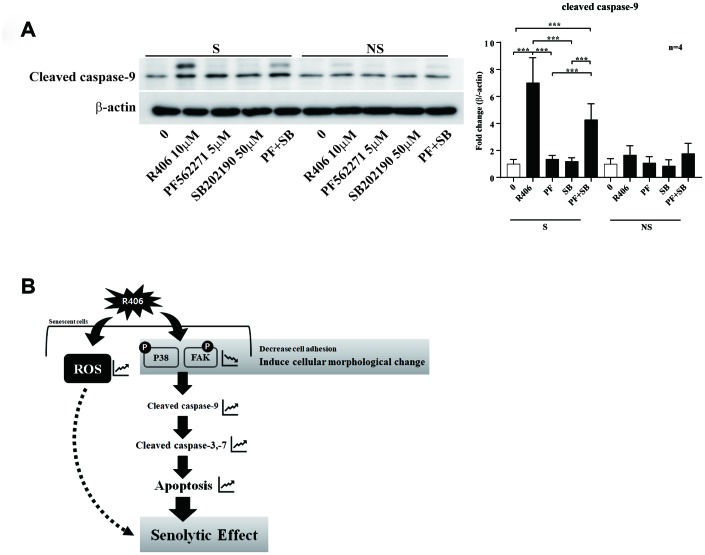
**R406 induces senolytic effects via blocking the phosphorylation of p38 MAPK and FAK simultaneously.** (**A**) Senescent (S) and non-senescent (NS) HDFs were respectively treated with DMSO, R406 (10 μM), PF562271 (5 μM), and SB202190 (50 μM) for one day and then western blot assay with anti-caspase-9 antibody was conducted to determine apoptosis. (**B**) Proposed mechanism of senolytic effect by R406. R406-induced cell death is mediated by inhibiting FAK and p38 activity as well as increasing ROS. *p<0.05, **p<0.01, ***P<0.001.

Although R406 induced FAK or p38 dephosphorylation, the inactivation of either kinase would not be enough to trigger apoptosis of senescent cells (Figures 5 and 6). Thus, we tested whether the R406- induced senolytic effect is mediated by the dual mechanisms of FAK and p38 at the same time. Indeed, the expression of cleaved-caspase-9 was induced only in senescent cells via co-treatment of PF562771 and SB202190 (Figure 7A). These results suggest that R406 induces apoptotic cell death of senescent cells by inactivation of multiple cell survival-related kinases including FAK and p38 MAPK.

## DISCUSSION

Selective clearance of senescent cells has been suggested to induce rejuvenation and longevity. In animal models, senolytic drugs have been shown to delay several age-associated disorders, to improve physical and cognitive function, and to extend lifespan [13, 14]. Because known senolytic drugs have limited diversity for their mode-of-action and affect change in a cell-type-specific manner [38], novel senolytic drugs are needed to improve efficacy and expend medical application against senescent cells.

This study demonstrated that R406 has several new aspects compared to other senolytic agents, beginning with its molecular mechanism for killing senescent cells. Resistance to apoptosis is one of the significant characteristics of senescent cells [2, 6, 7]. It is known that this resistance is associated with failure to suppress Bcl-2 activity [39] and, consequently, many senolytic agents target the Bcl-2 family-mediated anti-apoptotic pathway in senescent cells [40]. The R406 induced apoptosis pathway led to the increased expression of cleaved-caspase-9, -7, and -3 (Figure 3). This action by R406 is similar to other senolytic agents such as ABT263 (Supplementary Figure 4), quercetin, and A1331852 [22, 41, 42]. In contrast, R406 has no significant effect on expression of the Bcl-2 protein family (Figure 2).

To determine molecular pathways for R406-dependent cytotoxicity in senescent cells, we considered signals for cell survival, including the cell adhesion and MAPK pathways. FAK is a focal adhesion-associated protein kinase involved in cellular adhesion and spreading process, cell migration, and cell survival [7]. FAK expression and its phosphorylation for focal adhesion formation increased in senescent HDFs [8, 9]. In contrast, blocking the FAK pathway increases the loss of substrate adhesion and induction of apoptosis of anchorage-dependent cells [10–12]. In our study, we observed that cellular morphological changes appeared from 5 h after R406 treatment (Supplementary Video 1). Phosphorylation at Tyr576/577 and Tyr397 of FAK in senescent HDFs was strongly inhibited by R406 compared to that in non-senescent HDF cells. Tyr397 of FAK is an auto-phosphorylation site for focal adhesion formation and Tyr576/577 is a Src-dependent phosphorylation site and related to apoptosis [8, 10]. We showed the inhibition of phosphorylation at Tyr397 and Tyr576/577 of FAK by R406 (Figure 5A), which indicated that R406 induces cellular morphological change and apoptosis via FAK inactivation. However, the FAK inhibitor (PF562271) alone did not induce either expression of cleaved caspase-3 or apoptotic effect in HDFs (Figure 5). These results imply that R406 would eliminate senescent HDF cells by weakening cell adhesion and inducing apoptosis through FAK inhibition, but R406-dependent FAK inactivation would not be the only trigger for inducing apoptosis in senescent cells.

Next, we checked whether R406 alters phosphorylation of JNK and p38. Among them, R406 strongly inhibited the phosphorylation of p38 in senescent cells, in contrast to its actions in non-senescent cells (Figure 6A and 6B). Similar to the FAK inhibitor, p38 inhibitor (SB202190) alone did not stimulate cleaved-caspase-9 and -3 (Figure 6C and 6D). This observation implies that R406-induced p38 dephosphorylation would also be associated with senescent cell death, but that p38 inactivation could not solely trigger apoptosis of senescent cells. Interestingly, co-treatment with the FAK inhibitor and p38 inhibitor resulted in the cleavage of caspase-9, specifically in senescent cells (Figure 7A). These observations together with R406-induced kinase inhibition allow us to propose a working model for R406 as a senolytic whereby the drug-induced cell death is mediated by inhibiting FAK and p38 activity as well as increasing ROS (Figure 7B).

In apoptotic cell death, various kinase pathways, including MAPKs and AKT, are involved. [31–34]. Not only that, FAK exists at the upstream or downstream of AKT signaling and MAPK pathway [43–45]. These mentioned kinases are related to anoikis which is apoptosis induced by inappropriate cell-matrix interaction [46]. Previous studies demonstrated that anoikis was suppressed via a degradation of Bim and Bad by enhancing FAK, ERK, and AKT [47, 48]. We confirmed that R406 inhibits phosphorylation of AKT, ERK, and FAK (Figures 5 and 6). Thus, we anticipated that inactivation of FAK/ERK/AKT by R406 would increase the amount of Bim and Bad. As expect, R406 significantly induced the amount of Bim. Particularly, Bim level was slightly induced by R406 in non-senescent HDFs, but not as much as the senescent HDFs (Figure 2C). The results imply that R406 might induce the activation of anoikis in senescent cells and further studies about the process are waiting to be carried out. In terms of drug effectiveness, R406 can broaden the spectrum of senolytics in that R406 is effective in the RS model of genetically unmodified primary cells. In previous studies, typical senolytic agents (dasatinib, quercetin, ABT263, A1331852, and A1155463) were primarily studied in ionizing radiation-induced senescent cells, one of SIPS models [17, 22, 40, 49, 50]. Although RS and SIPS models share many common age-related cellular phenotypes like cell-cycle arrest, SA-β-gal induction or SASP increase, they also have distinct characteristics including patterns of protein expression [5]. Senolytic effects also present differently with senescence types [51]. Notwithstanding the limitation to verify the effectiveness of R406 in other cell types rather than HDFs as well as in an *in vivo* system, R406 could expand options for selecting effective combinations of senolytic drugs in various senescence contexts.

In conclusion, we demonstrate that R406 is a new class of senolytics that inhibits multiple cell survival signaling including FAK and p38 MAPK, which have been previously unrecognized in senolytic pathways. With diverse molecular mechanisms for eliminating senescent cells, R406 will be more effective in combination with senolytics targeting other anti-apoptotic pathways in senescent cells, which would become a valuable tool to overcome age-associated impairment.

## MATERIALS AND METHODS

### Cell lines and culture condition

Human diploid fibroblasts (HDFs) were obtained from the ATCC (PCS-201-010, Manassas, VA, USA). Cells were cultured in the growth media composed of DMEM (Welgene, Korea) supplemented with 10% FBS (Welgene), Antibiotic-Antimycotic solution (100 units/ml penicillin, 100 ug/ml streptomycin and 250 ng/ml amphotericin B, Welgene) at 37 °C in 5% CO_2_ incubator. We used senescent cells of which the population doubling time (DT) is more than 14 days and non-senescent cells of which DT is less than one days [25]. We used SIPS models of which non-senescent HDFs were treated with 20 μM etoposide for two days, or treated with 400 μM H_2_O_2_ for six days.

### Drug preparation

Drugs were purchased from the following: NVP-BHG712 (S2202), AZD (S7106), CCT129202 (S1519), Nintedanib (S1010), R406 (S2194), Axitinib (S1005), Bosutinib (S1014), selumetinib (S1008), Navitoclax (ABT263, S1001), PF561772 (S2890, FAK inhibitor), and SB202190 (S1077, P38 inhibitor) from Selleckchem (Houston, TX, USA). Staurosporine (S9300), etoposide (E1383), and H_2_O_2_ (216763) from Sigma-Aldrich (St. Louis, MO, USA). They were prepared 10 mM stock solution in DMSO. They were added in culture medium to obtain a suitable working solution. As a vehicle, DMSO as the same volume was added in culture medium. The stock solution was stored at -20 °C.

### Cell viability assay

Cell viability was determined using water-soluble tetrazolium salt-based D-plus cell viability and cytotoxicity assay reagent (CCK-1 reagent, Dongin LS, Korea). Cells were seeded in the 96-well plate and respectively treated with 0.5, 1, 5, 10 and 20 μM of NBP-BHG712, AZD and CCT129202, and 1, 2, 5, 10 and 20 μM of nintedanib, R406 and axitinib, and 0.1, 0.5, 1, 2 and 4 μM of bosutinib, and 1, 10, 25, 50, and 75 μM of selumetinib for 24 hours in the growth media**.** Cells were treated with 0.1, 0.5, 1, 5, and 10 μM of ABT263 respectively for one day or three days. Cells were then treated with 10 μl CCK-1 reagent and incubated for 4 hours at 37 °C in 5% CO_2_ incubator. After incubation, plates were read absorbance in 450 nm using the multi plate reader (Infinite M200PRO). In to check cell viability using the Hoechst 33342 staining, cells were seeded in the 24-well plate and respectively treated with 1, 2, 5, 10 and 20 μM of R406 for 24 hours in the growth media. Cells were then fix with 100% methanol for 10 min and washed with PBS. The Fixed cells were stained with 1 μg/ml Hoechst 33342 (#639, Immunochemistry Technologies, MN, USA) for 1 h and washed twice with PBS. Cell number was determined by measuring fluorescence intensity in Excitation/Emission: 355 nm/460 nm using the multi plate reader (Infinite M200PRO).

### SA-β-gal staining

To detect β-galactosidase activity, cells were stained using the senescence β-galactosidase staining kit (Cell Signaling, #9860) by following manufacturer’s instructions. Development of blue color by β-galactosidase staining was estimated under a microscope.

### Flow cytometry

Drug treated cells were harvested by trypsinization and resuspended in 1X Annexin V binding buffer (10 × Annexin V binding buffer: 0.1 M HEPES (pH 7.4), 1.4 M NaCl, 25 mM CaCl_2_) containing 5 μl of Annexin V-Alexa fluor®350 conjugate (Thermo Fisher Scientific, A23202) and 2 μl of 100 μg/ml propidium iodide per 1x10^5^ cells in 100 μl, and then incubation for 15 min at the dark. After the incubation, add 400 μl 1X Annexin-binding buffer (final vol. 500 μl). The cells were analyzed by flow cytometry within 1 h.

### Western blot analysis

Protein lysates were prepared by resuspending cell pellets in 2X laemmli sample (#161-0737, Bio-Rad Laboratories, Hercules, CA) buffer containing 5% β-mercaptoethanol. The protein lysates were separated by electrophoresis on SDS-PAGE and then electro-transferred onto Polyvinylidene difluoride (PVDF) membranes (Millipore Corp., Bradford, MA, USA). Detection of specific protein was carried out by enhanced chemiluminescence following the manufacturer’s instructions (P90720, Millipore corporation, MA, USA). We used the following primary antibodies: Caspase-9 (#9503, #7237), Caspase-8 (#9746), Caspase-7 (#8438), Caspase-3 (#9664), Bak (#3814), Bax (#2772), Bcl-xl (#2762), p-AKT (#4058), AKT (#2967), p-ERK (#4376), ERK (#4695), p-P38 (#9216), P38 (#8690), p-JNK (#9255), JNK (#9258), p-SYK (#2710), and SYK (#2712) were purchased from C*ell Signaling* Technology, *Inc*.. PARP-1 (sc-74470) and β-actin (sc-47778) were purchased from Santa Cruz Biotechnology, Inc.. p-FAK(Y576/577) (ab76244), p21 (ab7960) were purchased from Abcam. p-FAK(Y397) (611722), FAK (611008), and p16 (551154) were purchased from BD Biosciences. Densitometry analyses were performed using Image J software.

### Time-lapse imaging

Time-lapse images were captured using Axio-Observer Z1 microscopy (Zeiss) under incubating in PeCon microscopy incubator (PeCon GmbH) at 37°C and 5% CO_2_. The cell images were captured with 10 × objective in every 20 min for about 1.5 days to monitor cellular morphological change event.

### Detection of ROS generation

Cell were incubated with 10 μM dihydrorhodamine 123 (DHR123, Biotium, CA, USA) for 1 h at 37°C and then washed twice with phosphate buffered saline (PBS), and ROS content was measured in excitation/emission: 500 nm/536 nm using a multimode plate reader (Infinite200 PRO, Tecan, Switzerland). To normalize ROS-derived fluorescence intensity with cell number, DNA content of the ROS measured cells was estimated. Cells were stained with 1 μg/ml Hoechst 33342 (#639, Immunochemistry Technologies, MN, USA) for 1 h and washed twice with PBS. Cell number was determined by measuring fluorescence intensity in Excitation/Emission: 355 nm/460 nm using the plate reader. ROS levels were calculated by normalizing DHR123-stained values with Hoechst 33342-stained value. In the analysis using flow cytometry, drug treated cells were harvested by trypsinization and re-suspended in complete growth media adding 10 μM DHR123. The cells were incubated for 1 h at 37°C followed by washing twice with PBS, and then analyzed by flow cytometry within 1 h. The mean ± S.D. from four replicates was determined for each experimental group.

### Statistical analyses

All data were analyzed using ANOVA with Graphpad Prism 7 software. Post-hoc analyses were completed with Tukey’s Multiple comparison test with p<0.05 significance. DATA represent mean ± S.D. (*p<0.05, **p<0.01, ***p<0.001).

## Supplementary Material

Supplementary Figures

Supplementary Table 1

Supplementary Video 1
