# Supplementary material for: Identification of SYK inhibitor, R406 as a novel senolytic agent
Source: Aging (Albany NY). 2020 May 7;12(9):8221–40. doi: 10.18632/aging.103135 (PMC7244031; doi:10.18632/aging.103135)
Supplement: Supplementary Figures [file aging-12-103135-s002..pdf]

SUPPLEMENTARY FIGURES

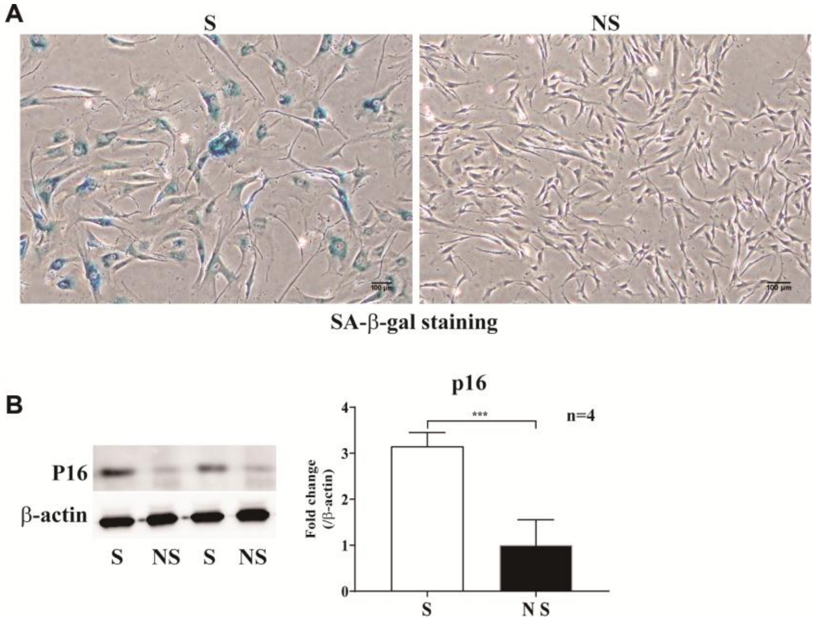

Supplementary Figure 1. Characterizing senescent and non-senescent HDFs. (**A**) Senescent cells increase SA-β-gal activity. Representative photomicrographs of SA-β-gal staining for non-senescent cells (NS) or senescent cells (S) are presented (scale bar: 100 μm). (**B**) Increasing p16 in senescent HDFs was determined by western blot analysis. **p<0.05, **p<0.01, ***P<0.001* compared to senescent HDFs.

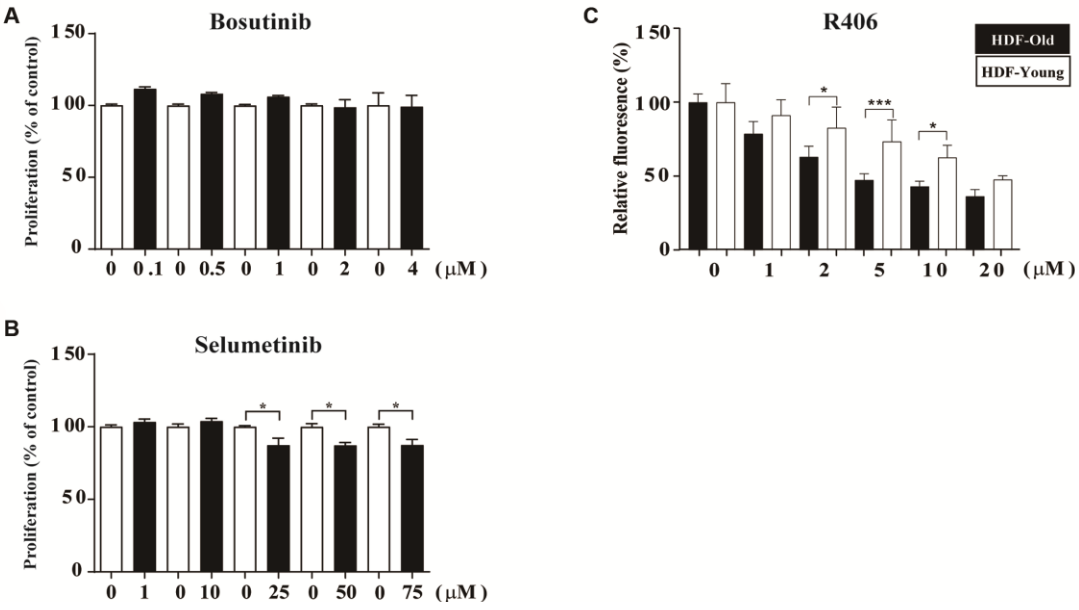

Supplementary Figure 2. Bosutinib and Selumetinib did not show senolytic effects in senescent cells. Senescent HDFs were respectively treated with DMSO (white bar), (**A**) Bosutinib (0.1, 0.5, 1, 2, 4 μM; black bar), and (**B**) Selumetinib (1, 10, 25, 50, 75 μM; black bar) for one day and then CCK-1 assays were conducted to investigate cell viability. (**C**) Senescent HDFs (HDF-Old) and non-senescent HDFs (HDF-Young) were treated with R406 (1, 2, 5, 10, 20 μM), and then, cell number was detected with Hoechst 33342 staining. n = 12, **p<0.05, **p<0.01, ***P<0.001.*

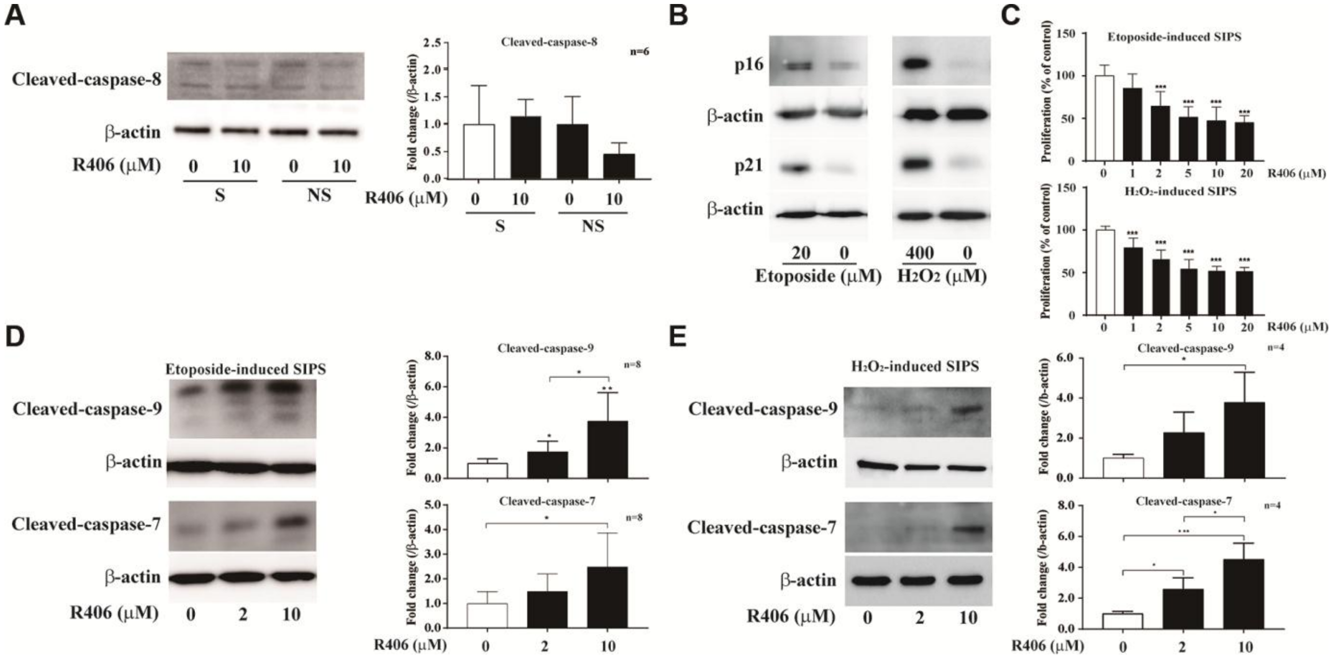

Supplementary Figure 3. R406 was not affect caspase-8 activity in senescent HDFs. (**A**) Senescent (S) and non-senescent (NS) HDFs were treated with DMSO and/or R406 (10 μM) for one day and then western blot assays were conducted with anti-cleaved caspase-8. (**B**) Non-senescent HDFs were treated with 20 μM etoposide for two days (left panel), and non-senescent HDFs were treated with 400 μM H_2O_2 for six days (right panel) and then western blot assays were conducted with anti-p16 and anti-p21. (**C**) 20 μM etoposide induced HDFs for two days were treated with DMSO and/or R406 (1, 2, 5, 10, 20 μM) for one day (upper panel), and 400 μM H_2O_2 induced HDFs for six days were treated with DMSO and/or R406 (1, 2, 5, 10, 20 μM) for one day (lower panel) and then CCK-1 assays were conducted to investigate cell viability. (**D**) Etoposide-induced SIPS were treated with DMSO and/or R406 (2, 10 μM) for one day and then western blot assays were conducted with anti-cleaved caspase-9, and -7. (**E**) H_2O_2-induced SIPS were treated with DMSO and/or R406 (2, 10 μM) for one day and then western blot assays were conducted with anti-cleaved caspase-9, and -7. *$p<0.05$, **$p<0.01$, ***$P<0.001$.*

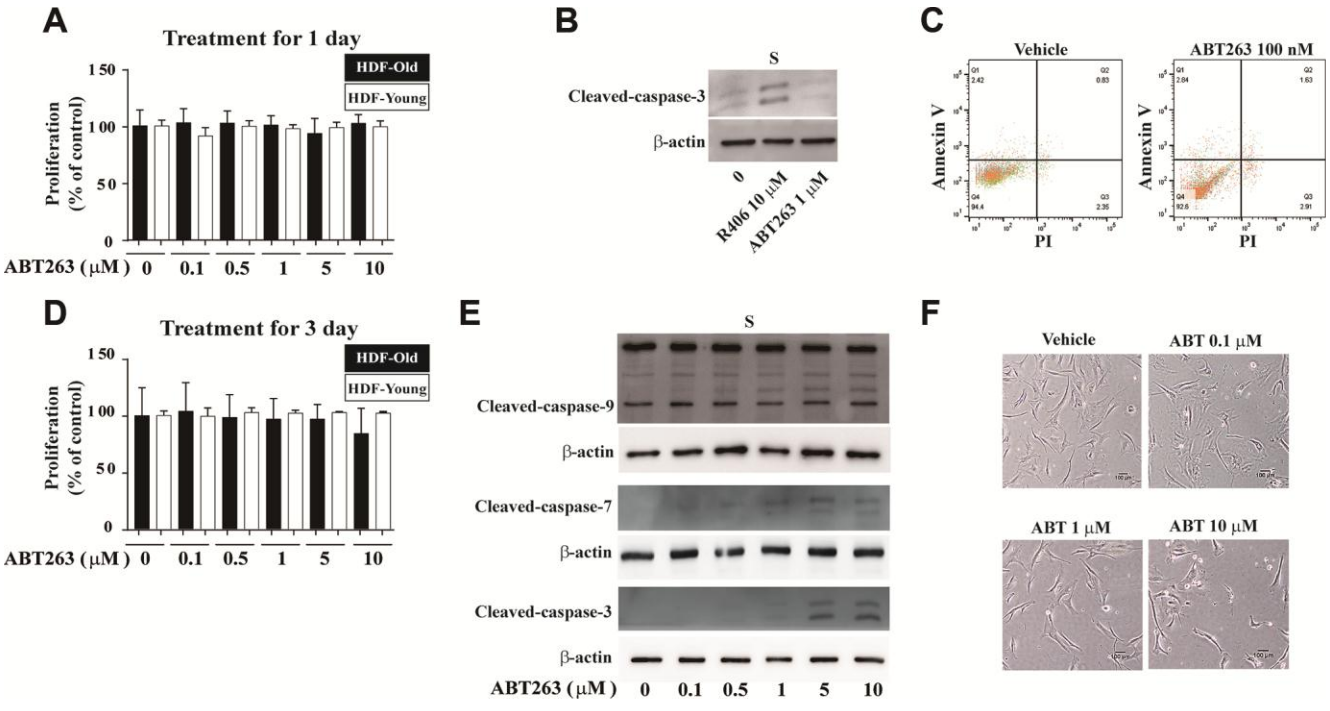

Supplementary Figure 4. ABT263 for one day treatment did not exhibit senolytic effect in the RS model. (**A**) Senescent HDFs (HDF-old) and non-senescent HDFs (HDF-young) were treated with DMSO or ABT263 (0.1, 0.5, 1, 5, 10 μM) for one day, and then CCK-1 assays were conducted to investigate cell viability. (**B**) Senescent HDFs were treated with DMSO or R406 (10 μM) or ABT263 100 nM for one day and then western blot assay (anti-caspase-3 antibody) was conducted. (**C**) Senescent HDF cell were treated with DMSO or ABT263 (100 nM) for one day and flow cytometry was conducted after Annexin V/PI staining. (**D**, **E**) HDF-old and HDF-young were treated with DMSO or ABT263 (0.1, 0.5, 1, 5, 10 μM) for three days. Then, (**D**) CCK-1 assays and (**E**) western blot assays (anti-caspase-9, anti-caspase-7, and anti-caspase-3 antibodies) were conducted. (**F**) Cell morphological change. Senescent HDFs were treated with DMSO or ABT263 (0.1, 1, 10 μM) for one day. Images were randomly captured by inverted microscopy (scale bar: 100 μm).

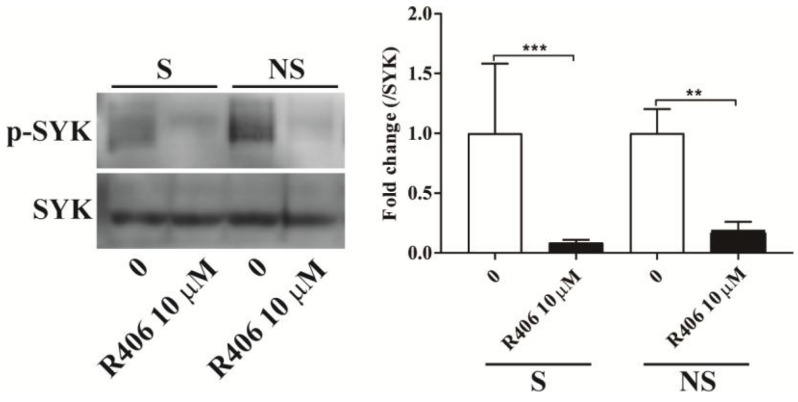

Supplementary Figure 5. R406 inhibited phosphorylation of SYK in both senescent and non-senescent HDFs. Senescent (S) and non-senescent (NS) HDFs were treated with DMSO or R406 (10 μM) for 1 h and then western blot assays with anti-SYK antibodies were conducted. *$p<0.05$, $^{**}p<0.01$, $^{***}P<0.001$.*
