# Supplementary material for: Identification of SYK inhibitor, R406 as a novel senolytic agent
Source: Aging (Albany NY). 2020 May 7;12(9):8221–40. doi: 10.18632/aging.103135 (PMC7244031; doi:10.18632/aging.103135)
Supplement: Supplementary Table 1 [file aging-12-103135-s001..pdf]

SUPPLEMENTARY TABLE

Supplementary Table 1. HTS results for selecting compounds that induce cytotoxicity or ROS levels in senescent HDFs.

Low proliferation			High ROS level		
**Cell and drug conc.**	**HGPS 5 µM**	**HDF 0.5 µM**	**Cell and drug conc.**	**HGPS 5 µM**	**HDF 0.5 µM**
**Drug name**	**ROS level**	**Proliferation**	**Drug name**	**ROS level**	**Proliferation**
OSI-027	0.94	0.686	Afatinib	2.25	0.838
Saracatinib	0.9	0.658	Dovitinib	2.48	0.908
SGI-1776 free base	1.24	0.657	Ponatinib	2.49	1.672
WYE-125132	1.16	0.653	CCT129202	3.64	1.267
PD184352	1.4	0.649	AZD5438	2.42	0.839
Bosutinib	1.66	0.648	IKK-16	2.37	0.886
Selumetinib	1.05	0.603	AZD3463	3.35	1.235
			GZD824	2.22	1.274
			BIO	2.38	0.882
**Low proliferation and Low ROS level**			**Low proliferation and High ROS level**		
**Cell and drug conc.**	**HGPS 5 µM**	**HDF 0.5 µM**	**Cell and drug conc.**	**HGPS 5 µM**	**HDF 0.5 µM**
**Drug name**	**ROS level**	**Proliferation**	**Drug name**	**ROS level**	**Proliferation**
LY2784544	0.88	0.678	Nintedanib	2.04	0.680
NVP-BHG712	0.75	0.677	Axitinib	2.1	0.619
R406 (Tamatinib)	0.81	0.645			
